# Experimental verification of the field theory of specific heat with the scaling in crystalline matter

**DOI:** 10.1038/s41598-021-97074-0

**Published:** 2021-09-13

**Authors:** Yuri Vladimirovich Gusev

**Affiliations:** Lebedev Research Center in Physics, Russian Academy of Sciences, Leninsky Prospect 53, str. 11 (38), Moscow, Russian Federation 119991

**Keywords:** Thermodynamics, Structure of solids and liquids, Scaling laws

## Abstract

The field (geometrical) theory of specific heat is based on the universal thermal sum, a new mathematical tool derived from the evolution equation in the Euclidean four-dimensional spacetime, with the closed time coordinate. This theory made it possible to explain the phenomena of scaling in the heat capacity of condensed matter. The scaling of specific heat of the carbon group elements with a diamond lattice is revisited. The predictions of the scaling characteristics for natural diamond and grey tin are verified with published experimental data. The fourth power in temperature in the quasi-low temperature behaviour of the specific heat of both materials is confirmed. The phenomenon of scaling in the specific heat, previously known only in glassy matter, is demonstrated for some zincblend lattice compounds and diamond lattice elements, with their characteristic temperatures. The nearly identical elastic properties of grey tin and indium antimonide is the cause for similarity of their thermal properties, which makes it possible to make conjectures about thermal properties of grey tin.

## Thermal theory as geometry

### Overview of the problem and its solution

In the present paper we continue the phenomenological study of the *geometrical theory of specific heat* of condensed matter^1–3^ based on the finite temperature field theory^4^. We explore further the scaling phenomena discovered in experimental data for the materials with the diamond and zincblend lattices^1^. In particular, here we verify the predictions made with this theory in Ref.^1^ for the low temperature behaviour of specific heat by using the statistical tools proposed in Ref.^2^ and available experimental data.

The new theory was called ‘the field theory of specific heat’ in Refs.^1,2^, because its mathematical formalism emerged from the deep modification and error correction of the finite temperature quantum field theory (QFT)^5,6^. Let us emphasize that the works^5,6^ were the big advance in the finite temperature QFT, yet they are erroneous, even within the scope of QFT, of course, along with the rest of literature on this subject. However, what we do is not even quantum field theory, since no quantum (or electromagnetic) fields are considered here. This physical model deals (so far) only with the elastic (sound or acoustic) waves in condensed matter. Furthermore, at its fundamental level, the model is built on the concept of a geodesic distance^4^, which belongs to geometry^7^. At its phenomenological level, the theory takes as the input parameters geometrical (mechanical) characteristics of a thermal system, the (average) inter-atomic distance and the velocity of sound in condensed matter, and gives as the output an observable quantity, specific heat function. It is reasonable then to call it a geometrical theory, after all geometry is physics.

The theory of specific heat of solid bodies was derived by P.J.W. Debye more than a century ago^8^, at the time of the advent of quantum theory^9^, but before the quantum field theory was created. The concept of four-dimensional space-time was already formulated^10^, but these mathematical ideas were not yet widely used in physics. Therefore, as explained in Refs.^1,2^, the Debye theory, and other models built upon it like the Born-von Karman theory of lattice dynamics, could not have been mathematically implemented at that time. Nevertheless, the Debye theory (or rather its elements) remains a standard model of condensed matter physics^11–14^. The Debye theory is usually presented as a model for the *lattice* specific heat. In reality, this model was derived for and is applicable to *any elastic media*: crystalline, amorphous and liquid matter.

However, the atomistic notion of discreteness of media was not properly built into the Debye model as explained in Refs.^1,2^. The main physical idea underlying the Debye theory, namely, the correspondence between the standing elastic (sound) waves in a medium and a medium’s heat capacity, could not be mathematically implemented at that time due to the lack of required mathematics. The early ideas of spectral geometry were developed by Peter Debye in his pioneering work^8^ and by Hermann Weyl^15^, but the evolution equation and its kernel were developed only in the second half of the twentieth century^16^. Debye was forced to make his model *closed* by postulating its equivalence to the discrete model of Einstein’s quantum oscillators^17^. However, this postulate cannot be verified experimentally, furthermore, it is *incompatible* with the model of elastic medium. This and other errors of the Debye theory rendered it practically useless. It fails to correctly describe matter’s specific heat in any temperature region, so, experimental physicists and engineers rely on data tables and fitting equations, e.g.^18^, instead of equations.

The new mathematical formalism for thermal theory let us solve a problem Debye posed a century ago. Namely, we found a *form* of the universal function that can describe the specific heat of different materials, crystalline and non-crystalline solids, as well as liquids. It is well known that Debye’s belief in a unique function of this type was wrong. However, we assert that abandoning his idea of the *scaling* altogether is wrong as well. As a matter of fact, the scaling of specific heats for glassy (non-crystalline) materials, well studied *empirically*, e.g.^19^, is one example of this principle.

In the present paper we demonstrate that the following scaling features, *predicted* by the field theory of specific heat, for *the same crystal lattice*, are supported by available experimental data for single crystals with the diamond and zincblend lattices:

1. specific heat functions of crystalline materials, with the same crystal lattice, exhibit the scaling, i.e. these rescaled functions coincide;
2. the magnitude of the specific heats at the corresponding characteristic temperatures, i.e. the ‘boson peak’, which indicates the threshold of the quasi-low temperature regime, is the same for materials with the same class of crystal lattice;
3. the slopes of the linear functions, in the quasi-low temperature regime, of the corresponding $C/T^3$ plots, are the same for the same class of crystal lattice.

In addition, we show that the proposed approach is viable by confirming its other predictions with newly acquired experimental data,

- the quasi-low temperature behaviour of the diamond type crystals of diamond and grey tin obeys the quartic in temperature power law, which is universal for any kind of condensed matter;
- the characteristic temperature of the grey tin’s specific heat, calculated previously from its transverse velocity of sound, as measured by the neutron scattering, is confirmed with experimental data.

We stress, that the focus of our work is on *mathematical equations*, with a few free parameters, that can explain properties of many condensed matter systems. Thus, one functional could quantitatively describe the specific heat of a whole class of crystal lattices, e.g. the diamond and zincblend lattices, if all its crystal anisotropy, through the stress tensor or equivalently the full set of velocities of sound, were taken into account. No other theoretical approach could achieve this. All existing models rely on extensive numerical simulations in a combination with numerous calibration parameters, which have no direct correspondence to physical observables.

We adopted the Debye’s idea about heat as the energy density of standing acoustic waves in elastic bodies and proposed its *pseudo-relativistic* implementation in the Euclidean (using the imaginary time variable) *D*-dimensional space-time^1^. Throughout this paper, the spacetime has four dimensions, as the real physical world, $D=d+1=3+1=4$, but the two-dimensional bodies and surfaces can be also described by this method^3^. This physical model uses the geometrical formalism for finite temperature field theory^4^. Therefore, the geometrical theory of specific heat is based on the new principles of theoretical physics, discovered after two decades of research in the field theory and gravity:

1. a generating functional of a physical theory is dimensionless and depends on a dimensionless variable;
2. dimensionless variables are composed of physical parameters (characteristics) of a medium;
3. this dimensionless functional is connected with a physical observable by a dimensional parameter (constant), determined (calibrated) by experiment.

### The geometrical formalism of the evolution kernel

The mathematical foundation and the derivation of the theory was published in the previous works^1,2,4^. Here we re-derive and explain the the mathematics of the evolution kernel, which remains little used in condensed matter physics. Let us start with the definition of the evolution kernel^16,20^, $K (s| x,x')$, as a solution of the *evolution equation*,

1 $$\begin{aligned} \Big (\frac{{\mathrm {d}}}{{\mathrm {d}} s} - \Box ^x \Big ) K (s| x,x') = 0, \end{aligned}$$

with the initial conditions,

2 $$\begin{aligned} K (s| x,x') = \delta (x,x'), \ s/\sigma (x,x') \rightarrow 0. \end{aligned}$$

The two-point *world function*, $\sigma (x,x')$, is a function that depends on two points in a spacetime with dimension *D*. The *proper time*, *s*, measured in square metres, parametrizes the *D*-dimensional *spacetime* interval; see for the details a book by Synge^7^. The proper time is a physical variable external to the spacetime coordinates^7,21^, it is neither a physical time nor a $D+1$ coordinate.

The *D*-dimensional Laplacian is defined as,

3 $$\begin{aligned} \Box \equiv g^{\mu \nu }\nabla _{\mu }\nabla _{\nu }, \end{aligned}$$

where $\nabla _{\mu }$ are covariant derivatives and $g^{\mu \nu }$ is the metric. We will skip all gauge field (electromagnetic) contributions below, although they will be needed for an electronic theory. Consequently, covariant derivatives of Eqs. (1) and (3) become ordinary derivatives.

The evolution equation, which is erroneously called ‘the heat equation’ in the quantum field theory literature, has nothing to do with the heat equation of mathematical physics. Let us emphasize the main features of this differential equation: (1) its derivatives act on a sought two-point function, which is a nonlocal kernel, (2) its solution is expressed in terms of the two-point world function and eventually in terms of geometrical and field tensors of a physical system, (3) the first order derivative is done over the proper time, which is an additional parameter, while the physical time is present in its operator and kernel.

The fundamental solution for the evolution kernel^7,16^ has a simple form,

4 $$\begin{aligned} K (s| x,x') = \frac{1}{(4 \pi s)^{D/2}} \exp \Big (-\frac{\sigma (x,x')}{2s} \Big ), \end{aligned}$$

where the spacetime dimension *D* enters explicitly only the pre-factor. For our physical applications, we need the functional trace of the evolution kernel that is defined as the integral, over the whole spacetime domain $\Omega$, of the kernel with the coincident points, *K*(*s*|*x*, *x*),

5 $$\begin{aligned} {\mathrm {Tr}} K(s)_{D} \equiv \int _{\Omega } {\mathrm {d}}^{D} x \, K(s|x,x) . \end{aligned}$$

The fundamental solution (4) is an *exact* result, which is the zero order term of various expansions. We will use this fact below.

The geometrical foundation for a new thermal theory^4^ is *defined* as a theory of the evolution kernel calculated in a spacetime with certain non-trivial topology. The *D*-dimensional spacetime with the Euclidean signature of the metric is a product of a compact *d*-dimensional (spatial) domain, ${\mathbb {R}}^d$, with the closed one-dimensional manifold, ${\mathbb {S}}^1$, whose circumference is $\beta$, measured in metres.

The trace of the evolution kernel on the manifold ${\mathbb {R}}^d \times {\mathbb {S}}^1$ can be calculated under the assumption that the *D*-dimensional evolution kernel (5) can be factorized into a one-dimensional temporal and a *d*-dimensional spatial parts^4^. We also use the fact that in flat space the world function^7^ is just a half square of the geodesic distance between two points, $\sigma (x,x')=(x-x')^2/2$. Then, its coincidence limit on a manifold ${\mathbb {S}}^1$ (a circle) is by the definition, $\beta ^2/2$, and the integral over ${\mathbb {S}}^1$ gives a factor of $\beta$—‘the volume’ of a one-dimensional space.

However, since ${\mathbb {S}}^1$ is a closed manifold, a geodesic can make several windings before the coincident limit (5) is taken (the loop is closed). We assume that any number of windings is allowed and take a sum over all such configurations:

6 $$\begin{aligned} {\text {Tr}}{K}(s|{\beta })_{1}= \frac{1}{(4 \pi s)^{1/2}} \int _{{\mathbb {S}}^1} {\mathrm {d}}^{1} x \, \sum _{n=1}^{\infty } \exp \Big (-\frac{(n(x-x'))^2}{4s} \Big )\Big |_{x=x'}= \frac{\beta }{(4 \pi s)^{1/2}}\ \sum _{n=1}^{\infty } {\mathrm {e}}^{-\frac{\beta ^2 n^2}{4s}} \end{aligned}$$

This sum resembles, but not equivalent to, the sum over thermodynamic ensembles in J.W. Gibbs theory. By the standard postulate^4^, the sum over these configurations is taken with equal weights. Therefore, the final expression for the *D*-dimensional ${\mathrm {Tr}} K(s)_{D}$ is expressed as^4^,

7 $$\begin{aligned} {\text {Tr}}{K}(s|{\beta })|_{D} = \frac{\beta }{(4 \pi s)^{1/2}}\ \sum _{n=1}^{\infty } {\mathrm {e}}^{-\frac{\beta ^2 n^2}{4s}} \ {\mathrm {Tr}} K(s)_{d}. \end{aligned}$$

We make now a crucial step by introducing *axiomatically* a new function $F({{\tilde{a}}|{\beta }})$ via the the proper time integral^1^,

8 $$\begin{aligned} -F({{\tilde{a}}|{\beta }}) \equiv {{\tilde{A}}} \int _{{\tilde{a}}^2/4}^{\infty }\! \frac{{\mathrm {d}} s}{s}\, {\text {Tr}} K(s|{\beta }), \end{aligned}$$

where the integral has a lower positive limit, in contrast to zero of Ref.^4^. This function is assumed to be defined up to an overall factor ${{\tilde{A}}}$, which would be determined by experiments, e.g. the measurements of elastic heat capacity of a condensed matter system.

The lower limit in Eq. (8) must be arbitrary positive, because both the integral’s measure (8) and the trace of the evolution kernel (5) do not exist at $s=0$. The value, ${\tilde{a}}^2/4$, has a factor 1/4 for further simplifications. Effectively, ${\tilde{a}}^2$ serves as a unit (a scale) of the proper time, *s*. Thus, we have two scales in a theory, $\beta$ vs ${\tilde{a}}$, which makes it possible obtain the theory’s functions expressed via a dimensionless variable. Both the integral measure and the integrand are dimensionless, which makes $F({{\tilde{a}}|{\beta }})$ *dimensionless* as well. Therefore, the term ‘free energy’ is not appropriate here.

In Eq. (7), we need the trace of the *d*-dimensional evolution kernel ${\mathrm {Tr}} K(s)$, which in three spatial dimensions^4^ is,

9 $$\begin{aligned} {\mathrm {Tr}} K(s)_{3} = \frac{1}{(4\pi s)^{3/2}}\ {\mathcal {V}}, \end{aligned}$$

where ${\mathcal {V}}$ is the volume of a spatial domain $\Omega$ of ${\mathbb {R}}^3$. This is the zero order term of the curvature expansion of the evolution kernel^20^. Let us stress that Eq. (9) is a generalization of a result that Debye^8^ and Weyl^15^ attempted to obtain. Even though they were successful with the discovery that a sought function is proportional to the domain’s volume, they could not possibly derive the proper time dependence in (9) since neither relativity theory, nor differential geometry were available yet. Therefore, Debye and Weyl resorted to the use of a frequency variable, intrinsically connected with time, that led to the inconsistent theory of specific heat.

The computation of (8) with (9) delivers the solution^1^,

10 $$\begin{aligned} -F (\alpha )_{3} = \frac{{\tilde{A}}}{\pi ^2} \frac{{\mathcal {V}}}{{\tilde{a}}^3} \sum _{n=1}^{\infty }\, \frac{1}{n^4 \alpha ^3}\Big (1 -\exp (-\alpha ^2 n^2) - n^2 \alpha ^2 \exp (-\alpha ^2 n^2)\Big ), \ \ \ d=3, \end{aligned}$$

of the dimensionless variable,

11 $$\begin{aligned} \alpha = \frac{\beta }{{\tilde{a}}}. \end{aligned}$$

Equation (10) is an exact dimensionless expression and the sum over *n* is expressed in pure numbers, $\alpha$. Thus, $F (\alpha )_{3}$ is not connected to any physical theory yet. All physics would emerge from the definitions for the Euclidean time, $\beta$, and the parameter, ${\tilde{a}}$.

Now we take the derivative of (10) over its variable $\alpha$ to obtain,

12 $$\begin{aligned} \frac{\partial F^{\alpha }}{\partial \alpha } = {\tilde{A}} \frac{3}{\pi ^2} \frac{{\mathcal {V}}}{{\tilde{a}}^3}\, \Theta (\alpha ), \end{aligned}$$

where,

13 $$\begin{aligned} \Theta (\alpha ) = \sum _{n=1}^{\infty }\, \frac{1}{ n^4 \alpha ^4} \Big \{ 1 -\exp (-\alpha ^2 n^2) - n^2 \alpha ^2\, \exp (-\alpha ^2 n^2) - \frac{2}{3} n^4 \alpha ^4 \, \exp (-\alpha ^2 n^2) \Big \}. \end{aligned}$$

**Figure 1:**
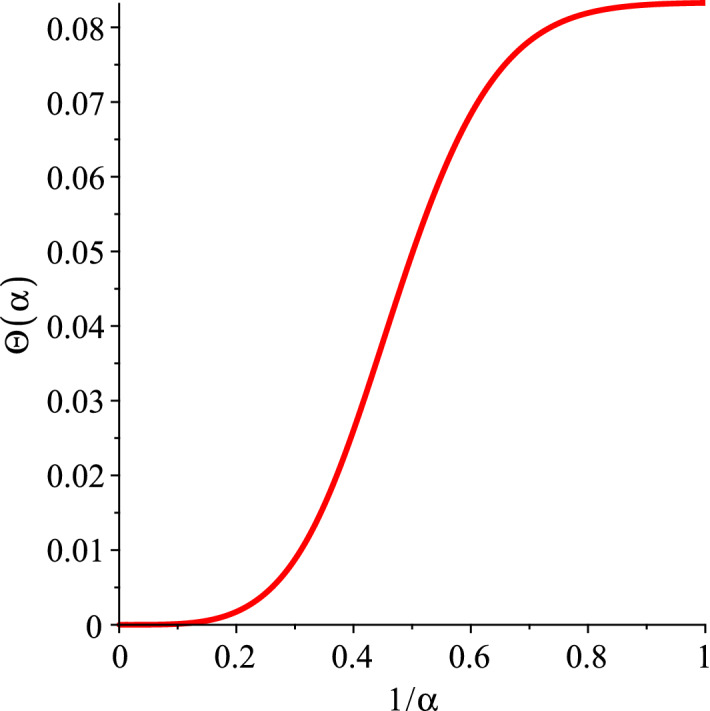
$$\Theta (\alpha )$$ versus $$1/\alpha $$, $$d=3$$.

The universal sum $\Theta (\alpha )$ presents the main function of the proposed formalism. Its maximum value in the asymptotic $\alpha \rightarrow 0$ is $\Theta (\alpha )_{\mathrm {max}} \approx 8.33 \times 10^{-2}$. $\Theta (\alpha )$ is not defined and therefore divergent at $\alpha =0$. Graphically, this function exhibits the behaviour shown in Fig. 1, where $\Theta (\alpha )$ is plotted as a function of the inverse variable, $1/\alpha$, for the needs of the following section.

The asymptotic of $\Theta (\alpha )$ at $\alpha \rightarrow \infty$ is the quartic power function,

14 $$\begin{aligned} \frac{\partial F^{\alpha }}{\partial \alpha } = {{\tilde{A}}} \frac{\pi ^2}{30}\, \frac{{\mathcal {V}}}{{\tilde{a}}^3}\, \frac{1}{\alpha ^4}, \ \alpha \rightarrow \infty . \end{aligned}$$

It is the limit (14) that will be used and explored with experimental data in the present work. All equations of the present section are universal, i.e. this is pure mathematics, which is universal. In the next section we will show how this mathematics could be used for building a thermal theory of matter.

### The geometrical thermal theory of the elasticity waves

There are several reasons for attempting to use the mathematics of the evolution kernel presented in the previous section for building a thermal theory of atomistic matter. The matter is assumed to be condensed in the present work, whether it could also be gaseous matter is being investigated at the moment.

First, it was discovered after decades of the development of finite temperature quantum field theory, see for a brief review Ref.^4^, that a circumference of the Euclidean time of a sub-manifold ${\mathbb {S}}^1$ could be associated with the inverse temperature of a thermal system. Restoring all the physical constants, this relation can be expressed in a form,

15 $$\begin{aligned} \beta = \frac{1}{B} \frac{h}{k_{{\mathsf {B}}}} \frac{v}{T}, \end{aligned}$$

where *B* is an experimental calibration parameter. The Planck constant $h= 6.62607015 \times 10^{-34}$ J s and the Boltzmann constant $k_{{\mathsf {B}}}=1.380649 \times 10^{-23}$ J K$^{-1}$ are exact^22^ according the Revised SI (2019) of physical units^23^. The characteristic velocity *v* used to be taken the velocity of light in vacuum, *c*, another defining physical constant, because quantum field theory is a relativistic theory. We make a reasonable generalization by assuming *v* to be a velocity that is intrinsic to a theory under consideration. Thus, for a thermal theory of atomistic matter it can be *a velocity of sound* in media.

The second idea comes from the history of partial differential equations that began from the wave equations finally reaching its covariant form (1) to (3). Therefore, it is natural to go back to the (non-relativistic) wave equation in three dimensions, $d=3$,

16 $$\begin{aligned} \frac{1}{v^2} \frac{\partial ^2}{\partial t^2} {\mathbf {u}}({\mathbf {x}},t) = \triangle {\mathbf {u}}({\mathbf {x}},t), \end{aligned}$$

where ${\mathbf {x}}$ is a three-dimensional coordinate, ${\mathbf {u}}$ is a vector of displacement, and $\triangle$ is a Dalambert’s operator. In order to work in a Euclidean spacetime defined above, we introduce the imaginary time $\tau$, this operation is also common in quantum field theory^4,16^. As a result the wave operator admits the form of the Laplacian (3),

17 $$\begin{aligned} \Box = \frac{1}{v^2} \frac{\partial ^2}{\partial \tau ^2} + \triangle . \end{aligned}$$

The third element of the proposed formalism is the method of the evolution kernel which tells us to seek a kernel of the evolution equation (1) instead of an acoustic spectrum of Eq. (16). Indeed, we are not really interested in knowing a specific form of the spectrum, but rather an integral function that characterizes it. As we saw in "The geometrical formalism of the evolution kernel" section, the evolution kernel required to be obtained as the functional trace presents a global geometrical function such as a volume or an area. This is a great reduction in complexity from working with explicit spectra.

Combining these three steps, we believe it is possible to solve the Debye’s problem in a completely different way that lets us avoid the self-contradictory calculation of Ref.^8^. Let us recall that the failure of the Debye theory of specific heat stems from the fact that thermodynamic temperature *T* could not be introduced into a mathematical model of the spectra of elastic waves. The model of acoustic waves was replaced with the model of quantum oscillators which were associated with the lattice nodes. As discussed many publications, the model of harmonic oscillators cannot be verified with experiment. For the present work it is sufficient to remember that the reason for the combination of these two models was a divergent integral over the frequencies of elastic waves at the high frequency limit^8^. However, this problem physically does not exist because the frequency spectrum is limited by the discreetness of condensed matter. In the method of the evolution kernel, we introduce the lower limit in Eq. (8), which is associated with the smallest wavelength determined by the average interatomic distance for amorphous matter or the lattice constant *a* for crystalline matter. Thus, the final definition of the new dimensionless variable is,

18 $$\begin{aligned} \alpha = \frac{1}{B} \frac{h}{k_{{\mathsf {B}}}} \frac{v}{a T}. \end{aligned}$$

The theory of specific heat with a single velocity of sound is calibrated by two parameters, *A* and *B*, which scale the sought function horizontally and vertically.

In Refs.^1,4^ we conjectured that the universal sum $F (\alpha )$ is the chief functional of a new thermal theory, built with geometrical analysis. The functional $F (\alpha )$ and its derivative over $\alpha$ are both dimensionless. This fact posed a fundamental problem of finding a way to obtain physical (dimensional) quantities, called observables, from a mathematical (pure number) expression. We used the only feasible way and introduced a dimensional factor with the *scaling postulate*^1^. The scaling postulate, states that the volume specific heat is determined by the derivative of (10) over its variable $\alpha$ with a factor with the physical dimensionality of the universal gas constant, $R = 8.314734$ J mol$^{-1}$ K$^{-1}$.

19 $$\begin{aligned} C_{{\mathcal {V}}} \equiv k_{{\mathsf {B}}} \frac{\partial F(\alpha )_{3d}}{\partial \alpha }. \end{aligned}$$

Observing that the pre-factor of $\Theta (\alpha )$ in Eq. (12) would be proportional to the total number of atoms, $N=\frac{{\mathcal {V}}}{{\tilde{a}}^3}$, we can find the molar specific heat (at constant pressure)^1^,

20 $$\begin{aligned} C = A k_{{\mathsf {B}}} N_{{\mathsf {A}}}\, \Theta (\alpha ), \end{aligned}$$

where the Avogadro constant is $N_{{\mathsf {A}}}=6.02214076 \times 10^{23}$ mol$^{-1}$. The overall numerical parameter *A* is different for different matter systems.

The molar heat capacity (20) is defined and measured at constant pressure, because the inter-atomic distance and the sound velocity change with changing pressure, but the theory’s input parameters that enter the variable $\alpha$ are assumed to be constant. The assumption that *v* and *a* remain approximately constant with the changing temperature is satisfied in the first approximation, as shown in Ref.^2^. This condition maybe violated in the pre-melting region due to thermal expansion and changing elastic moduli, but we do not consider this limit here. The expression (20) is a contribution from a single velocity $v_i$ supported within elastic medium: the proposed theory is not completed yet and it suffers the same shortcoming as the original Debye theory.

The inverse of the variable $\alpha$ is proportional to thermodynamic temperature due its definition (18). Thus, it is not a surprise that its behaviour shown in Fig. 1 resembles the Debye function. There are of course crucial differences. At sufficiently low temperatures, function (13) simplifies to power-like contributions. In this limit, which we called the *quasi-low temperature* regime^2^, only the slowest velocity of sound dominates the total specific heat. For diamond lattice materials this velocity is $v_5$ determined by $c_{44}$ elastic modulus^1^, for glassy materials it is the transverse velocity of sound^2^.

The quasi-low temperature behaviour of specific heat^2^ is the large $\alpha$ asymptotics of the function (20) gives the corresponding asymptotics of the molar specific specific heat,

21 $$\begin{aligned} C \propto k_{{\mathsf {B}}} N_{{\mathsf {A}}} \frac{1}{\alpha ^4}, \ \alpha \rightarrow \infty . \end{aligned}$$

As seen from the definition (18), this behaviour predicts the *fourth power* of temperature, $T^4$. The quartic in temperature contribution (21) in the total specific heat was already found in some experimental data sets in Refs.^1,2^. In the present work, we verify conclusions made in Ref.^1^ for the diamond lattice materials and supplement it with the analysis of some zincblend compounds.

From the dimensionless (scaling) property of the universal thermal function (13) of the dimensionless variable $\alpha$ we concluded^1^ that the threshold for the change in the functional behaviour can be identified from the characteristic dimensionless value,

22 $$\begin{aligned} \alpha _{0} = \frac{h}{k_{{\mathsf {B}}}} \frac{v}{a T_0}. \end{aligned}$$

which should be the same for materials of the same lattice class. Note, that the notation $\alpha _0$ was introduced in Ref.^2^ to replace the notation $\theta$ in Ref.^1^ in order to avoid confusion with the $\Theta (\alpha )$ function and to reflect its proper meaning.

Different values of the characteristic temperature $T_0$ can be then calculated with the known atomic and mechanical properties of a material and used to *empirically calibrate* specific heat functions. In other words, the *hypothesis* is that specific heat of only one material should be measured, while other functions of the same lattice class could be described by the same mathematical function, whose actual values can be found once $T_0$ for a material not measured is calculated from (22). The value of the specific heat at the characteristic temperature, $C_0$, is expected to be the same for all materials in the same lattice class. This procedure, when the theory is completed and calibrated, could greatly reduce the amount of experimental work needed. In Ref.^2^ we explained that the characteristics (22) is similar to or even coincide with some other characteristics introduced in different methodological approaches.

The Debye temperature, which originally was believed to be a universal characteristic of a specific matter^11^, became simply another form for presenting the measured specific heat which depends on temperature. At the same time, it is a convenient test for the analysis of the power behaviour of molar heat capacities. To indicate the fact that it is merely a testing function of experimental data, we removed all unessential numerical factors and defined it as^1^,

23 $$\begin{aligned} T_{\theta } \equiv T \left( \frac{R}{C}\right) ^{1/3}. \end{aligned}$$

It can be used to find the characteristic temperature, $T_0$, in the same way the standard graph of $C/T^3$ versus *T* is used. But the latter graph is more convenient for finding numerical values.

## Experimental verification and scaling

The characteristic temperature $T_0$ and the parameter $\alpha _0$ for natural diamond, derived from the experimental data obtained by J.A. Morrison’s group^25^, were different from the ones for other carbon group elements^1^. The power law of diamond’s specific heat in the quasi-low temperature regime was not determined. It was conjectured that higher precision measurements of natural diamond’s properties could produce new values that would be in agreement with the characteristics of silicon and germanium. The same characteristics for grey tin, $\alpha$-Sn, were theoretically derived from its elastic properties, rather than determined from the specific heat data, thus they should be verified. The quasi-low temperature behaviour of the diamond lattice and zincblend materials should be quantitatively similar, as was shown with GaAs. The experimental verification of these predictions is the subject of the next section.

### Physical properties of the diamond lattice materials

Thermal properties of the diamond lattices supported the idea of scaling, which axiomatically emerged from the field theory of specific heat^1^. The studied materials were three elements of the carbon group, diamond (C), silicon ($\alpha$-Si) and germanium ($\alpha$-Ge), and a chemical compound, gallium arsenide (GaAs). We reproduce here from^1^ the table of the physical properties of these materials, Table 1, with the addition of other compounds with the zincblend lattice: indium antimonide (InSb), gallium antimonide (GaSb), and indium arsenide (InAs).

In Table 1 the parameters predicted in Ref.^1^ are replaced by measured values, while parameters that are not directly measured are marked in italic. We added the columns for the specific heat, $C_0$, at the characteristic temperature $T_0$ and the scaled coefficient ${\tilde{d}}_1$ of the fitting equation (24), as discussed in "Scaling in the specific heat data of the diamond lattice elements and the zincblend compounds" section.

**Table 1:** Physical properties of the carbon group elements and the zinc-blend compounds.

Material	a	$c_{11}$	$c_{12}$	$c_{44}$	$\rho$	$v_5$	$T_0$	$\alpha _0$	$C_0$	${\tilde{d}}_1$	$T_{\mathrm {m}}$	$C_{\mathrm {DP}}$
Units	Å	GPa	GPa	Gpa	g/cm${}^3$	km/s	K	–	J/(K mol)	J/(K mol)	K	J/(K mol)
Diamond	3.567	1080.8	125.0	578.9	3.512	11.66	*161*	*1.55*	*1.25*	*0.88*	–	*29.0*
$\alpha$-Si	5.431	165.8	63.94	79.63	3.329	4.674	39.4	1.67	1.189	1.42	1685	29.16
$\alpha$-Ge	5.657	128.5	48.26	66.80	5.3256	2.746	21.4	1.73	1.133	1.40	1210	28.76
$\alpha$-Sn	6.489	66.7	36.5	30.2	5.7710	*1.62*	12.0	1.59	*1.176*	*1.03*	*800*	*28.0*
InSb	6.489	66.7	36.5	30.2	5.771	1.62	11.0	1.73	1.015	1.25	800	28.00
GaSb	6.096	88.3	40.2	43.2	5.614	2.07	15.0	1.73	1.033	1.24	985	28.38
InAs	6.058	83.4	45.4	39.5	5.68	1.83	14.0	1.65	1.047	1.25	1215	
GaAs	5.653	118.8	53.7	59.4	5.32	2.47	21.0	1.67	1.187	1.25	1513	29.08

The value for the lattice constant of natural diamond is from Ref.^26^, while diamond’s elastic constants and velocity are from Ref.^27^. These measurements were later confirmed by Ref.^28^. The values derived from the specific heat data of the zincblend compounds in Table 1 are from Ref.^29^. The actual measurement of the elastic moduli of GaAs was made in work^30^.

It is important to note that the molar specific heats of chemical compounds here and in Ref.^29^ are given *per number of atoms*, as fundamental constituents of condensed matter, not per number of molecule, i.e. one mole is equal to $N_{{\mathsf {A}}}$ of atoms even for a material with many-atomic molecules. Therefore, data taken from some other references should be divided by 2 to make them consistent with this rule.

As different from Ref.^1^, table 1 contains the melting temperature, $T_m$, not a general critical temperature, which previously included the ablation of diamond and the lattice transformation of grey tin. The melting of diamond occurs at temperatures approaching 5000 K, under high pressure^31^, so we leave this cell blank.

According to the field theory of specific heat, the *slowest* velocity of sound, which is one of transverse velocities, dominates the specific heat at low temperature^1^. For the diamond lattice, it is the transverse velocity $v_5$, in the notations of Ref.^27^. The universal link between the transverse velocity of sound and the low temperature behaviour of specific heat of glasses was discovered long time ago, e.g.^32^. This is an example of the same phenomena we continue to study here with the diamond and zincblend lattice data.

In Ref.^2^, the linear fit to the scaled specific heat function $C/T^3$ was introduced,

24 $$\begin{aligned} C/T^3 = d_0 + d_1 T, \ T<T_0. \end{aligned}$$

Obviously, this form implies the presence of a $T^3$ contribution in specific heat, which must exist due to the surface heat capacity, as hypothesised in Ref.^1^. Regretfully, the topic of surface specific heat was excluded from the final version of Ref.^2^ (yet mentioned in the analysis of the fitting function (24) on p. 10) on the request from an anonymous referee (see the publication’s history and its earlier versions in arxiv.org). This exclusion caused misunderstanding of the field theory of specific heat among condensed matter experts. This fact reflect the negative side of the journal peer reviewing process.

The consideration of a condensed matter system as a spatial domain with a smooth boundary, existing in the four-dimensional space-time with the cyclic Euclidean time, is a core mathematical concept of the finite temperature field theory^4^. The boundary (a surface for a 3-d body or an edge for a 2-d sample) of a condensed matter system determines its physics. The surface specific heat is a dominant contribution at temperature lower than the quartic power (quasi-low temperature) regime. But even in the quasi-low temperature regime^2^, the absolute value of quartic function (21) may be comparable to other contributions, which are always present in experimental data, as is shown below. Furthermore, the full function of the specific heat is the exponential sum (13), and we only consider the leading contribution of the $v_5$ velocity, while other modes do contribute as well.

The fact that there is always a cubic contribution present in the specific heat (24) means that first statistical estimates done in Ref.^1^ were not complete. Is is clear that even if one were to expect the specific heat behave strictly as a $T^4$ function, this function should have the form $C=a+(T-b)^4$ because the origin of this quartic function is not at $T=0$, which is a forbidden temperature value in the finite temperature field theory^4^. Then, this would be a full polynomial of the fourth order, with a constraint on its coefficients. Without the complete theory of specific heat yet, the following combination can be used as an approximation,

25 $$\begin{aligned} C = d_3 T^3 + d_4 T^4, \ T<T_0. \end{aligned}$$

It is consistent with (24), but not equivalent to it. The coefficients of (24) and (25) could coincide only within statistical uncertainty, $d_0 \approx d_3$ and $d_1 \approx d_4$, because the fitting is done respectively to scaled and original specific heat data.

For silicon and germanium, which were considered in Ref.^1^ (but only the germanium’s analysis was reported), we used the data from work^33^ and treated them with the above fitting equations. Silicon gives the coefficients $d_1 = 5.89(20) \times 10^{-7}$ J/(K$^{5}$ mol) and $d_0 = 2.3(4.2) \times 10^{-7}$ J/(K$^{4}$ mol). The standard errors are shown in the round brackets, i.e. $5.89(20) \equiv 5.89 \pm 0.20$. The very large standard error for $d_0$, whose range of acceptable values includes zero, means that the $T^3$ contribution is negligible. The coefficients for germanium came up as $d_1 = 6.68(30) \times 10^{-6}$ J/(K$^{5}$ mol) and $d_0 = -6.64(4.25) \times 10^{-6}$ J/(K$^{4}$ mol), which is confronted with $d_4 = 6.01(37) \times 10^{-6}$ J/(K$^{5}$ mol) and $d_3 = 3.41(5.97) \times 10^{-6}$ J/(K$^{4}$ mol). Again, statistical significance of the coefficient of $T^3$ is not satisfactory due to the errors, and we put $d_3$ to zero. This means, according to the field theory of specific heat, that experiments measured really bulk properties of the substances, and the surface heat was small. Indeed, the work^33^ says that single crystal specimens were broken into pieces with the average size of 3 mm.

### Testing the theory with grey tin data

Like carbon, silicon and germanium, tin is also an element of group IV of the Mendeleev’s periodic table of chemical elements, whose 150th anniversary of the discovery was celebrated in 2019. Apart from allotropes created at high pressures, two forms of tin exist at pressures and temperatures available outside a lab. White tin ($\beta$-tin) is a metal with the body-centred tetragonal (bct) lattice structure. At temperature lower than 286.4 K it turns into semiconductor, grey (gray) tin ($\alpha$-tin) with the cubic lattice of the diamond type^34^. This transition is commonly known as ‘tin pest’, a damaging factor in technological applications^35^.

While thermal properties of $\beta$-tin are well studied^36^, $\alpha$-tin’s specific heat remains poorly measured. Nevertheless, the acoustic frequencies of grey tin lattice were determined in the neutron scattering experiments^37^. Incidentally, the frequencies measurements^38^ done with the neutron scattering were fitted with 10 second-neighbour parameters of the Born-von Karman model. This large number of parameters raises a question about the validity of the postulate on next neighbour interactions in a lattice let alone the *descriptive* nature of the lattice dynamics.

In Ref.^1^ we used these experimental data to derive thermal properties of grey tin. The *implied* slowest velocity of sound was derived as $v_5 \approx 1.68\times 10^3\,$ m/s. Then, the characteristic parameter $\alpha _0 \approx 1.73$ (denoted by symbol $\theta$ in Ref.^1^) for silicon and germanium was assumed to be valid for $\alpha$-tin as well. These numbers together with the lattice constant of $\alpha$-tin gave the characteristic temperature $T_0\approx 11$ K.

These values for $\alpha _0$ and $T_0$ were called in Ref.^1^ predictions, but it turned out they were *postdictions*, because we overlooked the old work^39^, where the specific heat of grey tin was studied at temperature from 7 to 100 K. That paper contains a small set of experimental data, which is reproduced in Table 2.

**Table 2:** Specific heat of grey tin, Ref.^39^.

*T* (K)	7.0	8.0	9.0	10.0	12.0	15.0	20.0	25.0
*C* (J/(K mol)	0.180	0.301	0.464	0.674	1.18	2.08	3.74	5.31
*T* (K)	30.0	40.0	50.0	60.0	70.0	80.0	90.0	100.0
*C* (J/(K mol)	6.69	8.91	11.17	13.4	15.5	16.9	18.2	19.5

Let us now use these data to test our theory. Hill and Parkinson^39^ had two different samples of the ‘coarse powder’ of grey tin. They measured the specific heat of a higher purity sample from 2 to 20 K. As clear from Table 2 and the corresponding curve, these measurements agree well with the higher temperature measurement, from 12 to 120 K, performed with a poorer quality sample. Our analysis requires the lower temperature set of Ref.^39^ which is, unfortunately, rather scarce.

**Figure 2:**
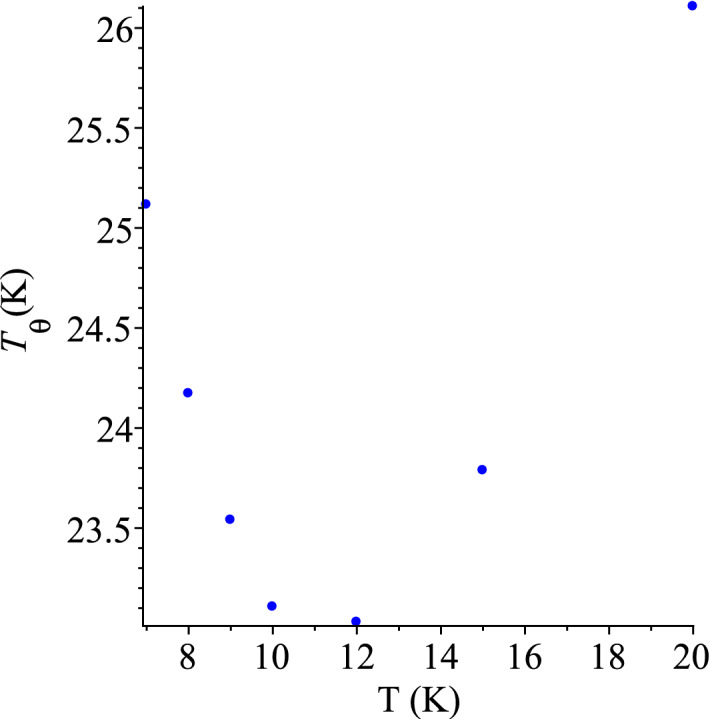
QLT regime of grey tin, Ref.^39^.

We can use either the graph of $C/T^3$ versus *T* or the plot of the *pseudo-Debye temperature* (23) to locate the characteristic temperature, $T_0$. If extracted from Fig.2 of its data, the lowest value of $T_{\theta }$ is 12 K. However, judging from the shapes of similar curves for other materials, true $T_0$ could be between 10 and 12 K, so, it is reasonable take the midpoint of 11 K. This would make a perfect coincidence with the value predicted in Ref.^1^, but it could be, of course, questioned. Nevertheless, 11 or 12 K is a close match any way, considering the large experimental uncertainty. The corresponding value of the dimensionless parameter $\alpha _0=1.73$ automatically agrees with the predicted one and it coincides with the value for InSb in Table 1. This further confirms the similarity of thermal-elastic properties of these two materials. There is no data point for T = 11 K in Ref.^39^, the average of two neighbouring points, 10 and 12 K, would give 0.92 J/(K mol), but we prefer to take this value from InSb again, $C_0=1.0$. This is another conjecture about the grey tin’s specific heat for future experiments.

Looking at the left, ascending branch of the graph in Fig. 2, we test the statistical hypothesis (albeit only with 4 data points), whether the corresponding data for $C/T^3$ versus *T* are governed by the function (24), or equivalently whether *C* in Table 2 for $T < T_0$ is governed by (25). Fitting of Eq. (24) to the data in Table 2 gives the values, $d_0 = 1.84(37) \times 10^{-4}$ J/(K$^{4}$ mol) and $d_1 = 4.96(43) \times 10^{-5}$ J/(K$^{5}$ mol). Needless to say, that with so few points statistical significance of the fit by a straight line is nearly perfect, with the $\chi ^2$-statistic close to zero and the *p*-value almost unity. This straight line (24) does not cross the axis origin, and it certainly should not, for the absolute zero of temperature is absent from our geometric formalism (18). This means that the quartic term is not the only term in Eq. (24) even in the QLT regime, let alone in the would-be-complete expression of the full theory. By fitting the function (25) to the data of Table 2, we obtained values $d_3 = 2.20(4) \times 10^{-4}$ J/(K$^{4}$ mol) and $d_4 = 4.56(43) \times 10^{-5}$ J/(K$^{5}$ mol). They agree with the the coefficients of Eq. (24), up to their statistical uncertainty, as expected.

### Making more predictions for $\alpha$-tin

It is disturbing that the elastic moduli of grey tin, measured in neutron scattering experiments^37^, as reproduced in Table 1, are very different from the ones given in standard chemistry reference book^40^. Apparently, the reference values which were calculated according to theoretical models should be considered *erroneous* in view of the experimental data^37^ and their consistency with the thermal properties discussed above.

In Table 1, it is striking to see that mechanical (crystalline and elastic) characteristics of $\alpha$-tin single crystals almost exactly match the corresponding values for indium antimonide, InSb. This remarkable fact was first noted in Ref.^37^. This is an instance of the phenomena of scaling explored in "Scaling in the specific heat data of the diamond lattice elements and the zincblend compounds" section. This matching offers a unique opportunity to verify the most valuable feature of the field theory of specific heat: we conjecture that the fully measured specific heat of $\alpha$-Sn mono-crystals should coincide with the corresponding function for InSb^37^.

In the literature, the transition from $\beta$-Sn to $\alpha$-Sn is described as a way to produce grey tin. However, all references which we found, e.g. cited in Ref.^41^, describe the opposite transformation, from the diamond lattice to the metallic tin, only in thin films of $\alpha$-Sn deposited on zincblend substrates. This raises the question, if melting of grey tin can occur in principle?

The work^36^ reported the specific heat of $\beta$-tin collected from different sources from 20.54 up to 300 K. Thus, we can ask, if the metallic form of tin could be studied below the $\alpha$-$\beta$-transition, why the diamond lattice tin would not be studied *above* this transition’s temperature? Indeed, the only data point determined at high temperature of 283.6 K, $C= 25.44$ J/(K mol), was measured nearly a century ago^42^, as reproduced in Table V of Ref.^43^, p. 481. It is known that crystalline seeds of $\alpha$-tin significantly change this transition^44^. This lattice transformation also occurs at different temperatures for thin films.

By employing the similarity of grey tin and indium antimonide we conjecture, that the proposed Dulong-Petit value in Table 1 really corresponds to the *melting* temperature of grey tin, which could be achieved for pure single crystals of $\alpha$-tin, isolated from other lattice allotropes. If this phenomenon could be observed, it would likely occur at temperature close to the melting temperature of InSb, $T_m =800\ K$. These are different chemical substances, but if our hypothesis that thermal phenomena, including phase transitions, are governed by elastic (acoustic) properties of matter, is true, this conjecture is viable and worth exploring experimentally.

New precision measurements of the specific heat of grey tin could deliver more numerical values to check the proposed functional form of *C*. The method of growing single crystals of grey tin used to be difficult^45^, but a simpler method is known now^46^ that can produce even shaped single crystals. We hope that this situation with the scarce data^39^ would encourage experimentalists to measure the thermal properties $\alpha$-tin. Such experiments are urgently needed for the reference texts used in experimental physics and technology.

### Revisiting the natural diamond data

It was a sheer coincidence that the first set of high precision data of specific heat we found, were those of the group IV elements^25,33^. These data gave an opportunity to test the new theoretical proposal. In Ref.^1^ preliminary statistical estimates of the characteristics of specific heat of diamond, silicon and germanium were made. There, we concluded that the data for natural diamond of Ref.^25^ (the measurements were done with 160 g of industrial quality diamonds with the average dimension of 3 mm) did not allow us to make a statistically significant selection between the two powers, $T^3$ versus $T^4$. However, we have re-analyzed these data with the extended statistical fit (25) proposed in Ref.^2^, and our conclusion is different now: the quartic power law clearly gives a contribution, in the quasi-low temperature range, from 80 to 130 K. A combination of two factors led to the earlier wrong statement: (1) the empirical choice of the temperature range which was too long (from 27 to 174 K); (2) a single quartic term in the fitting equation was used instead of Eq. (25). This once again shows that the surface specific heat must be always taken in account when analysing data in the QLT regime.

**Figure 3:**
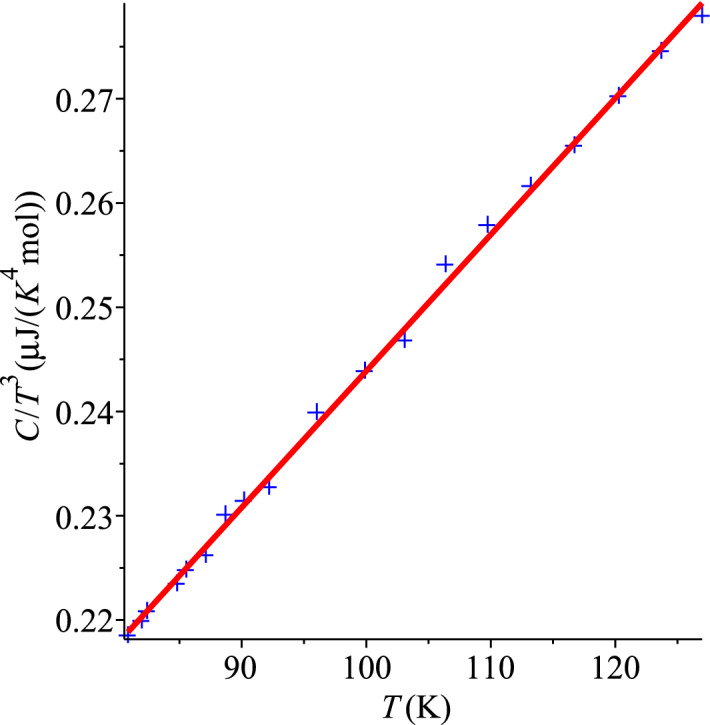
QLT regime of natural diamonds, Ref.^25^.

As one can see, the graph of $C/T^3$ versus *T* for natural diamond in Fig. 3 is as good as other similar plots. The ansatz (25) is fitted to the raw data of Ref.^25^ with the coefficients $d_3=1.155(16) \times 10^{-7}$ J/(K$^{4}$ mol) and $d_4=1.286(15) \times 10^{-9}$ J/(K$^{5}$ mol). The linear fit (24) of $C/T^3$ gives similar values, $d_0=1.130(13) \times 10^{-7}$ J/(K$^{4}$ mol) and $d_1=1.308(13) \times 10^{-9}$ J/(K$^{5}$ mol). These sets of values agree with each other, within standard errors. This shows good statistical significance of the hypothesis (25). It is obvious that the graph in Fig. 3 is nearly a straight line.These coefficients also show that at about 100 K both terms, cubic and quartic, give contributions to the total specific heat, with comparable absolute values. It means that the surface specific heat should always be taken into account (see also Ref.^3^). The complete field theory of specific heat should be calibrated, which is a task for future.

We confirm the above conclusion with the higher precision data, reported (but not published) in Ref.^47^. The work^47^ produced an excellent data set, which has 368 data points for *C* between 28.26 and 280.30 K. This study explored the dependence of diamond’s specific heat on isotope content for three different isotope combinations. The technology of producing artificial diamonds introduces iron atoms up to 0.15 mol% of diamond, and this contamination can change diamond’s thermal properties^47^. Therefore, we refrain here from using artificial diamond data and leave the isotope dependence for a later study.

The diamond’s thermal characteristics deviated from those for other two elements, silicon and germanium^1^. Specifically, the characteristic temperature was determined as 173.3 K, which gave the value $\alpha _0=1.44$, different from 1.73 for the other elements. In Discussion section of Ref.^1^, it was conjectured that the low temperature thermal properties of diamond should really be similar to those of other elements with the diamond type lattice. In fact, diamond has the widest known range of the quasi-low temperature regime among all chemical elements (this range is probably comparable to zinc-blend compounds with similar mechanical properties, e.g. cubic boron arsenide (BAs)^48^). This wide temperature range makes it difficult to assign a specific value to $T_0$. Instead of leaving the old value of $T_0$, we decided to trust the scaling emerging in the data and conjectured that it could really be determined via the specific heat $C_0$. Looking at its values for the carbon group elements in Table 1 we extrapolated this sequence for diamond. Then, the characteristic temperature could be found from the data of specific heat in Ref.^25^, it came out $T_0 = 161\ K$. The new $T_0$ produces the new dimensionless parameter $\alpha _0= 1.55$, which seems to continue the decreasing trend along the element’s column.

**Figure 4:**
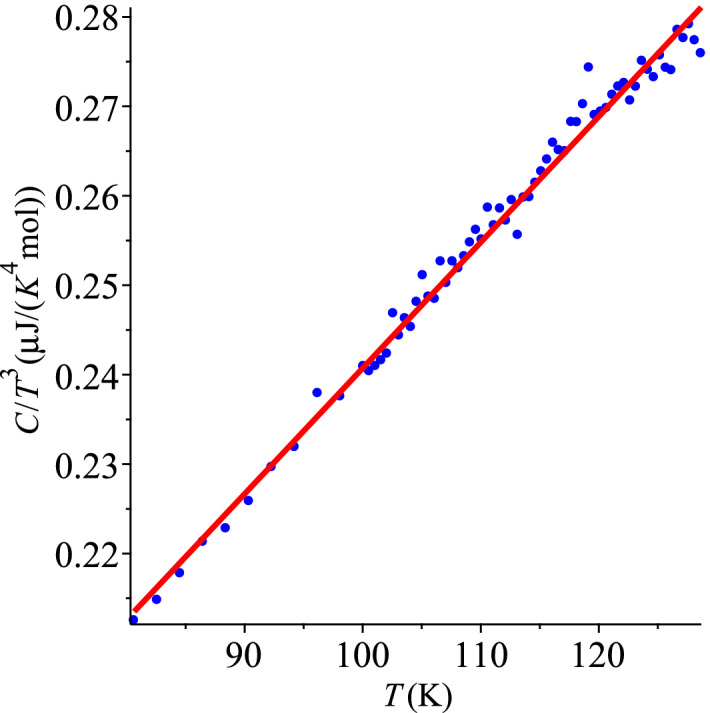
Specific heat of a 48.1 mg natural diamond, unpublished data for Ref.^47^.

The ansatz (24) is fitted to the data of Ref.^47^, which are presented in Fig. 4, with the coefficients $d_0=1.001(21) \times 10^{-7}$ J/(K$^{4}$ mol) and $d_1=1.407(19) \times 10^{-9}$ J/(K$^{5}$ mol), while fitting (25) to the specific heat *C*, recovered from $C/T^3$, gives $d_3=1.070(30) \times 10^{-7}$ J/(K$^{4}$ mol) and $d_4=1.346(26) \times 10^{-9}$ J/(K$^{5}$ mol), for a gem quality natural diamond of mass 48.1 mg (0.24 carats). Using this mass and the diamond density, it is easy to estimate the average dimension of a stone. For an uncut stone it is fair to approximate its shape by a sphere, which gives a value of about 3 mm, same as for the diamonds used in Morrison’s study^25^. This means that their effective sizes (specific surfaces) were comparable, and this fact is reflected in the values $d_0$ ($d_3$) which are close. Without more detailed information about the samples of each work^25,47^, we cannot investigate whether an apparent difference in these coefficients is caused by the difference of effective sizes or experimental uncertainties.

**Figure 5:**
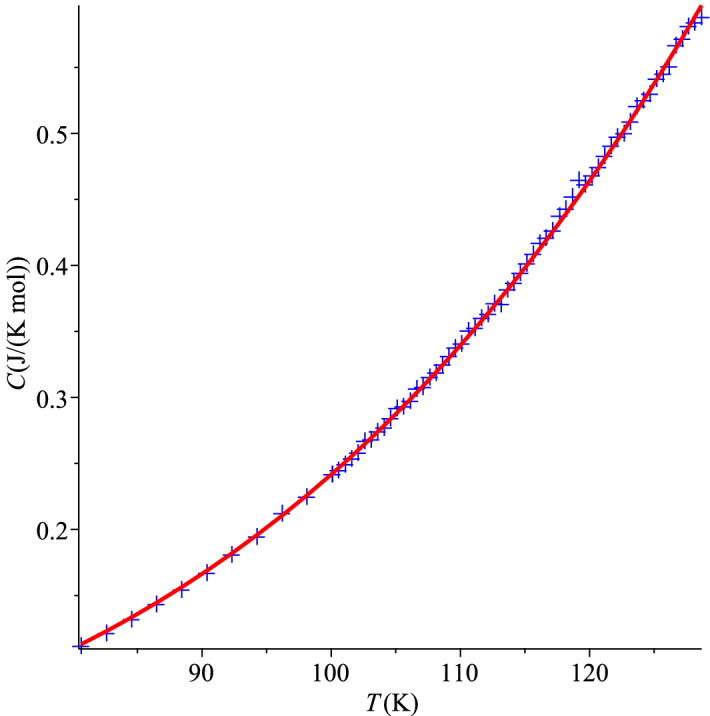
QLT behaviour of the specific heat of natural diamond, unpublished data for Ref.^47^.

Summarizing, two different data sets, obtained at two different labs, with different samples, produced very close values that characterize the specific heat of natural diamond, including the surface specific heat. The result of fitting the data shown in Fig. 4 with (25) is displayed in Fig. 5.

Natural diamond was the first material whose specific heat was used to test the Einstein’s model based on the Planck’s quantum theory^17^. It was *the worst* possible choice though, because of the very high characteristic temperature of diamond, i.e. its temperature of the QLT threshold. Besides, the specific heat of diamond was not measured with needed precision for another forty years, and even after that it was confusing to determine the right power behaviour, as Refs.^1,2^ have shown. Let us stress once again, the work^17^ is entirely erroneous, as explained in *all* condensed matter textbooks. Yet, all textbooks begin with the introduction of this model, which leads to its fairly wide use in the ‘centaur’ type models of specific heat.

The problem of the $\alpha _0$-parameter of natural diamond remains, one contributing factor is the large surface heat capacity of the diamond samples, as discussed in Ref.^3^, which is less significant for the silicon and germanium measurements considered in Ref.^1^. Besides, the elastic properties of diamond seem to be different from other elements of group IV. Namely, silicon^49^ and germanium^50^ have negative thermal expansion coefficients (contraction of a crystal) at some low temperatures. However, no negative thermal expansion, i.e. thermal contraction, was detected in experiments with diamond^26^. In our model, thermal expansion is important to take into account^2^ because it changes the volume and the lattice constant, which enters the dimensionless variable $\alpha$. Perhaps, these are the reasons natural diamond could not fit to the scaling curve discussed in "Scaling in the specific heat data of the diamond lattice elements and the zincblend compounds" section.

### Scaling in the specific heat data of the diamond lattice elements and the zincblend compounds

The phenomenon of scaling has been studied empirically for a long time in the physics of glasses (see some refs. in Ref.^2^). The same phenomenon occurs in crystalline matter, but it is less spectacular due to anisotropy of crystals and larger surface heat of samples. The phenomenon of scaling can be exposed by the parameter $\alpha _0$, which should be (almost) the same for all materials within the lattice class. This conjecture is not proven for the elements, but it is better supported by the zincblend compounds as seen in Table 1.

The calibration of the geometrical theory of specific heat^1^ requires two experimental parameters, *A* and *B*. Parameter *A* determines the scaling in a vertical direction (the ‘height’) of the specific heat graphs for a given group of materials. In general, it could be fixed by the Dulong-Petit value, $C_{DP}$, at the phase transition temperature, but $C_{DP}$ is conjectured to be the same for all materials in the same lattice class. Another special value of specific heat is $C_0$ at the characteristic temperature $T_0$, which indeed is the same for the zincblend lattices and nearly the same for the diamond lattices in Table 1. Parameter *B* , which governs the horizontal scaling (the ‘width’), can be determined with the characteristic temperature, $T_0$. Therefore, according to the revised Debye scaling hypothesis which we adopt here, specific heat functions in the same lattice group are scaled by $T_0$, i.e. if temperature is made dimensionless as $\tau =T/T_0$, the functions should coincide, i.e. form the universal curve (‘master curve’ as called in the physics of glasses).

**Figure 6:**
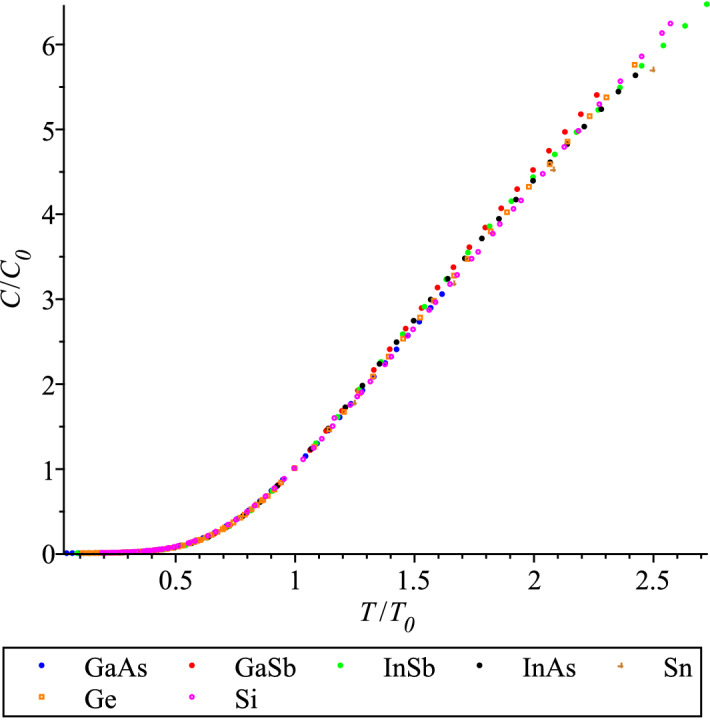
QLT regime of the rescaled specific heats of the diamond and zincblend lattice materials.

**Figure 7:**
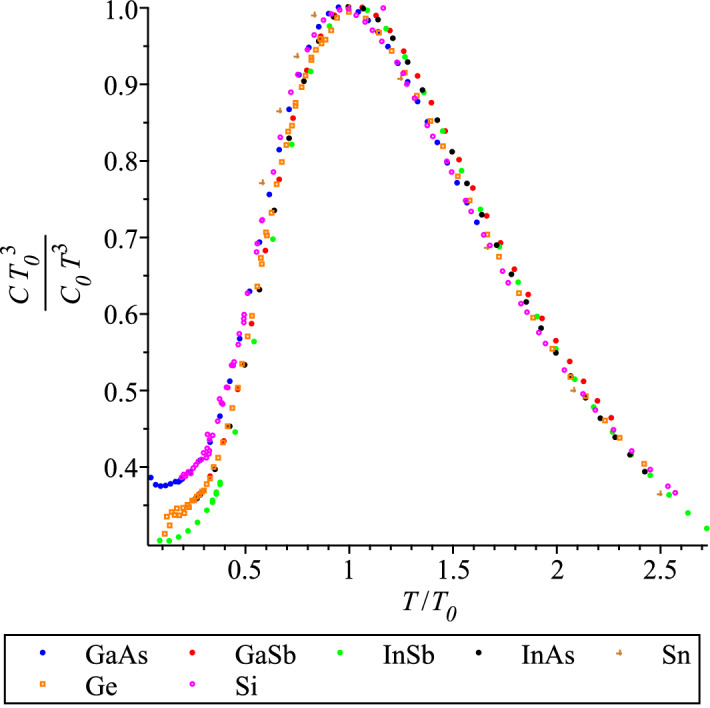
QLT regime of the rescaled $C/T^3$ graphs for the diamond and zincblend lattice materials.

We introduce the scaled parameter,

26 $$\begin{aligned} {\tilde{d}}_1= d_1 \cdot {T_0}^4, \end{aligned}$$

(the dimensionality of $d_1$ is J/(K$^{5}$ mol)). As seen from Table 1, the scaled coefficients ${\tilde{d}}_1$ for all diamond lattice materials nearly coincide. This fact is certainly not accidental, it is a feature of the scaling phenomena: specific heat functions of all materials in the same crystal class should be the same function, and the material specific characteristic, in this case $T_0$, allows to make up a material specific function. Because the range of temperatures, at which the specific heats of zincblend compounds are measured, vary we cannot produce the master curve at higher temperatures. However, the similarity of the specific heat functions looks convincing in the graphs scaled with their characteristic values, like in Figs. 6 and 7. The form of the maxima displayed in Fig. 7 is dubbed in the condensed matter literature ‘boson peak’ and used to be associated with glassy materials only. However, this is a universal feature of *any* condensed matter system, and this fact is confirmed by more and more experimental studies of crystalline matter, e.g.^51,52^.

Therefore, the scaling here can be only approximate in principle because of the surface specific heat, which depends of the sizes and shapes of samples, see Ref.^3^. In Figs. 6 and 7 the surface heat capacity, which behaves as $T^3$ at low temperatures, is visible as the left tails of different magnitudes. The best coincidence is observed for a set of zincblend compounds of Ref.^29^ because that work used large crystals and therefore its measurements are closer to the so-called ‘bulk’ specific heat.

Let us stress again that the crucial difference of the scaling, observed in Figs. 6 and 7, for a specific class of crystal lattice, is that the maxima $C_0$ do not require the rescaling, i.e. dividing by their maxima, as they do for glassy systems, because all $C_0$’s are expected to coincide, see Table 1, after clearing away all experimental uncertainties *and the size effects*. The latter procedure was not done here, for further discussion of the surface heat capacity see the future publication based on Ref.^3^. We also insist that a researcher should always be aware that any visualization can only be indicative and often confusing, and it should not be relied upon in the development of a physical theory.

**Figure 8:**
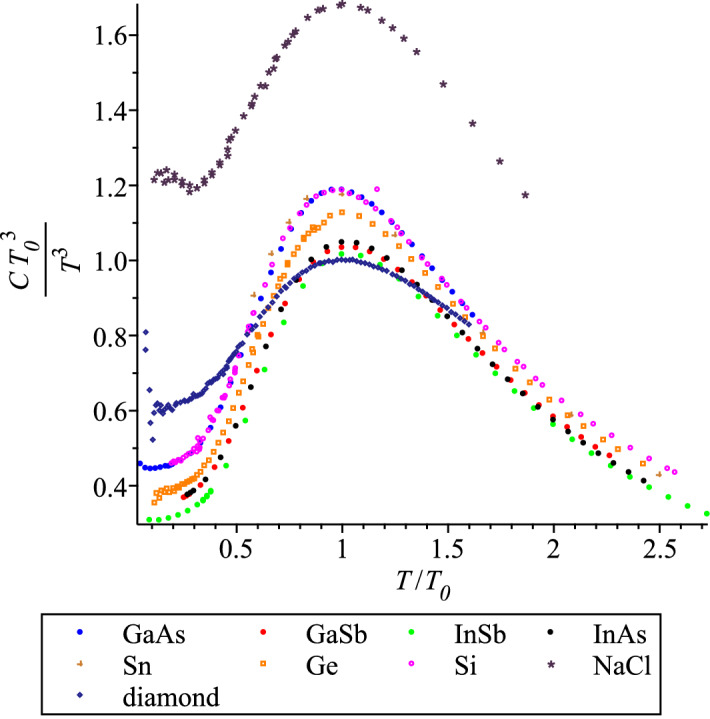
QLT regime of the $C/T^3$ graphs for the diamond and zincblend lattice materials, plus NaCl, without rescaling.

To have an unbiased view of the discussed phenomenology, let us look also at these graphs without rescaling, $C/C_0$, Fig. 8.

In order to clearly demonstrate that the scaling feature unifies materials with the same lattice class, we plotted in Fig. 8 the specific heat of sodium chloride, NaCl, by the data from Refs.^53,54^ (discussed in more detail in "Confrontation of the Debye theory with experiment" section below). NaCl also has a cubic lattice, but not of a zincblend class, therefore, its set of acoustic velocities is different. We remind that the lattice constant and the slowest velocity of sound determine the characteristic temperature of a material according to Eq. (22). Thus, even with a relatively big dispersion of the maxima of specific heats in Fig. 8, sodium chloride is an outsider. We leave it to other researchers to explore the scaling within a crystal class that NaCl belongs to.

We also plotted the troublesome diamond in Fig. 8, by the data from Ref.^25^. The main trouble with diamond is its very high surface capacity, as discussed in detail in Ref.^3^. The surface contribution is relatively high not only in the QLT regime, i.e. to the left from the $C_0$ point, but also at higher temperatures. This experimental fact is well seen in this figure. Nevertheless, we believe that we achieved the main aim of this section by showing that the scaling phenomenon within a crystal class, the limited version of Peter Debye’s dream^8^, is present in principle, further investigation is required.

## Summary

- The previously calculated thermal characteristics of grey tin are confirmed by available experimental data, up to experimental and statistical uncertainties.
- The specific heat function of $\alpha$-Sn is expected to match the specific heat of InSb, including the melting temperature, when direct measurements would be made with single crystals of pure grey tin.
- The specific heat of natural diamond behaves similarly to other diamond lattice materials. Its QLT asymptotic contains the fourth power of temperature, as confirmed by two independent data sets.
- The scaling in the specific heat data of zincblend compounds is exhibited by the universality of these functions, scaled by their characteristic temperatures.

## Discussion

### Confrontation of the Debye theory with experiment

The critical analysis of the Debye theory^2^, made *after* the results of the field theory of specific heat were axiomatically derived^1^, showed that this textbook model is theoretically inconsistent and experimentally erroneous. The similar critique of the Debye model was also done by other authors, e.g.^55,56^. Its key predictions, the cubic temperature law of the specific heat at ‘low’ thermodynamic temperatures and the universal limiting value of specific heats at melting temperatures are incorrect. Experimental data exhibit the linear in temperature growth of specific heat beyond the traditional Dulong-Petit value of $3R \approx 24.93$ J/(K mol) adopted from thermodynamics of dilute gases. The specific heat of solid state matter reaches at melting temperatures the limiting value of 27–29 J/(K mol), which is different for different materials. The behaviour of specific heat in the quasi-low temperature regime contains the universal quartic term, with the addition of a sample specific cubic contribution of the surface heat capacity. However, the key ideas of P. Debye, the scaling expressed as a universal function of specific heat and the velocity of sound as the main physical characteristic of heat, were correct. The field theory of specific heat^1^ presents the mathematically correct implementation of these physical ideas.

False theories that have never been verified are presented in textbooks, despite the experimental progress with acquiring precision data of specific heat. We discussed in Refs.^1,2^ the misleading graphical proofs of the Debye theory presented in the very influential textbook of Kittel^11^. Let us address here another textbook, a monograph by M.T. Dove on the lattice dynamics^57^. There, a comparison with experiment, based on the specific heat of sodium chloride, NaCl, is made with help of Fig. 5.5 on p. 76. The book claims that plotted experimental data (no publication reference is given) displays the cubic in temperature behaviour of the specific heat at low temperatures, therefore, this graph is supposed to experimentally prove the cubic law of the Debye theory.

We discuss here only the Debye theory because, the lattice dynamics of Born-von Karman does not predict any observable physical quantities, like the specific heat, it only fits its internal parameters (atomic interactions between nodes of a lattice) to the experimental data of acoustic frequencies^57^, usually determined by the neutron scattering. Criticism of the theory of lattice dynamics was presented in theoretical^2,55,58^ and experimental^59,60^ works.

**Figure 9:**
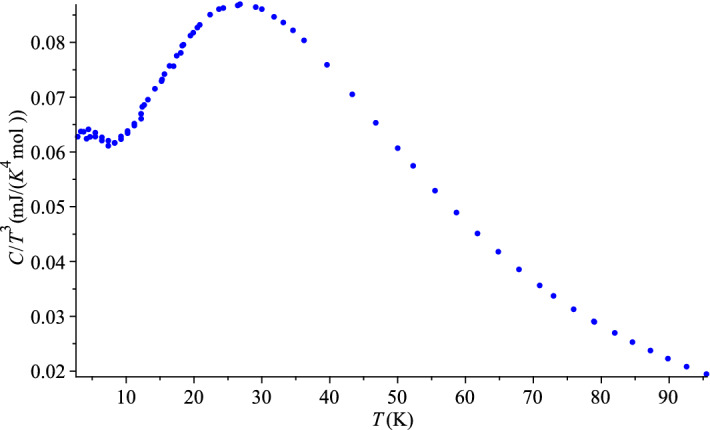
Specific heat of sodium chloride, data of Ref.^54^ as presented in Ref.^53^.

We searched for publications on the specific heat of NaCl, some sources are cited and discussed in Ref.^3^, but here we consider the study done in the lab of Morrison^54^. Together with D. Patterson he measured the specific heat of sodium chloride for the range of temperatures from about 3 to 267 K. However, in that paper they presented only the analysis and the graph for these experiments. The actual experimental data were published much later in the appendix of Ref.^53^. These data represent a typical set of specific heat behaviour, as seen in the graph of $C/T^3$ versus *T*, Fig. 9, where no cubic power law, which should be the *horizontal* straight line, is present, except at the very low temperature tail, which was already discussed. In Fig. 9 the cubic law behaviour in the range of temperatures from 8 to 20 K, claimed in the textbook^57^, is not observed. The approximately cubic behaviour at $T < 10\ K$ is an evidence of the non-universal surface specific heat.

The origin of this confusion in the literature on specific heat is clear: one may fit a data set with *any* fitting function and obtain some fitting coefficients, but one must always verify the statistical significance of the proposed function by calculating its statistic to select the best fitting function. Therefore, if raw data are treated, we can fit them with different power-law functions or their combinations, but different functions would have different statistical significance, as quantified by statistics like $\chi ^2$ and standard errors.

The limit on the specific heat at higher temperature appears due to the cut-off imposed on the acoustic frequencies spectra of condensed matter by a finite inter-atomic distance^1^. Condensed matter as ideal elastic media is a mathematical over-idealization, instead, matter has discrete, atomistic structure. This physical restriction limits the wavelengths of elastic waves in condensed matter. Properly incorporating this limit into a theory, as done in the field theory of specific heat, can resolve main troubles of the Debye theory.

The high temperature limit, at the melting temperature, of the Debye theory is fixed to the value of 3*R*, which is taken from the thermodynamics of gases. The vast experimental evidence demonstrate that the equipartition theorem is often not valid even in the heat theory of gases. Thus, it is not correct to consider it as the universal law and use it beyond its original scope of applications, dilute gases. The specific heat functions grow beyond this limit, e.g.^56,61^.

Anomalous ‘deviations’ from the Debye cubic law at low temperature, Chap. 2 of Ref.^13^, are not corrections to the Debye theory, they are indications of its failure. The proof of this failure is the demonstration, with already available experimental data, of the fact that the cubic in temperature contribution of the specific heat is *not* universal, i.e. it is not a ‘bulk’ property of the given material, but it depends on a size and a shape of the body via its effective size. This is shown in Ref.^3^ with the specific heat of rock salt powders and natural diamonds. The dedicated precision measurements of substances with various geometrical characteristics are urgently needed. Perhaps, reference data for specific heats should be measured again in view of this experimental evidence.

### Physical theory versus mathematical model

The first two publications on the field theory of specific heat^1,2^ met the criticism of R. Pässler, summarized in the dedicated paper^62^. It is encouraging to see the early interest in these ideas since one of the goals of our publications was to excite the critical revision of existing thermal theories by condensed matter physicists^1^, p. 71. Despite its generally negative opinion, the empirical analysis^62^ extends the exploration of experimental data we began in Refs.^1,2^, and its conclusions generally agree with the field theory of specific heat.

The preliminary statistical analysis of Ref.^1^ was improved in Ref.^2^. In fact, the work^62^ used the form of the statistical estimate, (24) and (25) introduced in our second paper^2^. Nevertheless, we emphasize that the focus on an exact and universal power of temperature in experimental data at the QLT regime is misleading. We used this subject to highlight the differences from the textbook theories and to draw the attention of the solid state physics community to the glaring problems in the condensed matter thermodynamics. For crystalline matter, which is anisotropic and support several longitudinal and transverse velocities of sound, the quartic law may be observed, among other terms, in a range of temperatures, we call the quasi-low temperature regime. The $T^4$ power is overcome by the cubic law of surface heat (25) below this temperature region and suppressed by the exponents of the universal thermal function (13) above it.

The completed and calibrated field theory will include all geometrical (volume, surface, edge) contributions to specific heat and be derived with the full set of group velocities of sound that may exist in a condensed matter body. For example, the surface specific heat, which is a $T^3$ function only in *its* QLT regime, but otherwise the universal thermal function of surface heat^3^ is qualitatively similar to the bulk one (13). The surface heat capacity contribution is present at any temperature, but its *relative* contribution with respect to the bulk specific heat depends on temperature. The numerical analysis of measured specific heats in Ref.^62^ supports this idea by finding ‘sub-quartic’ and ‘super-quartic’ behaviours. These behaviours may be determined by the full spectrum of acoustic frequencies of a crystal that was not incorporated yet into the field theory of specific heat. At the same time, the phenomena of surface heat capacity also must always be taken into account as shown above and in Ref.^3^, as it is obviously responsible for the polynomial (25), which may be more complex due to several transverse velocities.

Regretfully, the work^62^ is devoted entirely to mathematical modelling, with the focus on the $T^3$ versus $T^4$ problem, and leaves theoretical physics out of consideration. The difference between the two is fundamental. A physical theory describes and *predicts* the functional behaviour of a physical quantity by a mathematical construction based on the input from observed physical quantities of *different kind*. For example, in the proposed geometrical formalism, the specific heat behaviour is derived from the sound velocities and the lattice constants, i.e. thermal properties are derived from mechanical ones. Instead the modelling is concerned with the best fit of *existing data* by mathematical functions that have no immediate relations to measured physical properties.

The modelling of specific heat by extended polynomials performed in Ref.^2,62^, was first introduced to the low temperature physics by Barron and Morrison^63^ in order to describe experimental data obtained in their laboratory, because existed theories could not account for the observed behaviour of specific heat. However, the proposed fitting equation was just empirical, it was not justified by a physical theory. The diatomic linear chain model considered in the appendix of Ref.^63^ cannot serve as a theoretical foundation for the proposed specific heat function for the same reasons the lattice dynamics cannot be accepted as a physical theory, as discussed above and in Ref.^2^. Therefore, some coefficients of such a polynomial are determined with unacceptably low statistical significance, i.e. the statistical hypothesis that the data correspond to a tested function is rejected. Unfortunately, sixty years ago physicists believed more in ‘fundamental’ theories and had less trust in statistical analysis.

Every year many works on the heat capacity of solid matter are published, yet they all assume that basic tenets of the Debye theory are valid. Despite this unreasonable belief, some of these publications do report finding the *fourth power law* in the specific heats at lower temperatures. For example, the quartic, $T^4$, behaviour of the specific heats below the characteristic temperatures (22), within the QLT regime, has been supported by the investigation^64^, which used entirely different tools and concepts. That study describes the numerical simulations of vibrational modes of stable glasses and concludes: “*Quasi-localized modes obey* $D_{\mathrm {loc}} \propto \omega ^4$.” Quasi-localized modes in the language of our formalism are the low frequency shear acoustic waves, specifically, the $v_5$ sound velocity for the diamond lattices. The quartic power of the frequency $\omega$ corresponds to the quartic power of temperature, if the Debye theory were valid and used, as it it was in the work^64^. Therefore, the authors of Ref.^64^ arrived at the partially right result. Partially, because glasses are not special. The field theory of specific heat predicts the quartic power law behaviour for all frequency contributions of a condensed matter system made of glassy, crystalline and liquid matter. Besides, in our previous paper^2^ we explained the confusion about counting the frequencies and other grave errors in the Debye physical model and its mathematical implementation, which led to a handicapped theory, that is being ‘repaired’ since then with various scaffoldings. Therefore, the work^64^ belongs to the class of physical models that fit the measured parameters by adding more free parameters to a theory, like a series of power for the inter-atomic interactions and does not predict any new phenomena.

In general, physical models, which are limited in the scope of their applications, may contain many more physical parameters, e.g. Standard Model of elementary particles^65^ or Standard $\Lambda$CDM (the cosmological constant with the cold ‘dark matter’) Model in cosmology^65^. However, the fewer parameters a model contains, the better its *predictive power* is. This is the reason these two modern theories are still called ‘models’ and massive efforts to improve and simplify them are under way.

Let us repeat that any scientific theory must not only describe existing but also predict new physical phenomena. Thus, in Ref.^1^ we made some predictions for the specific heat of grey tin using only its known *mechanical* properties and *thermal* properties of other materials in the same lattice class. These predictions are tested in "Testing the theory with grey tin data". Even though the grey tin data were already published, they were not known to us, so this case could be considered a blind calibration test. Likewise, the contradiction of the QLT behaviour of natural diamond was resolved with more experimental data and better statistical tools, *not* by amending the theory.

### Current state and future completion of the theory

Here our final aim is, as was Debye’s^8^, to create a theory that can describe thermal properties of many materials at once, and by doing so to predict ones that are not measured yet. The physical meaning of the scaling is the mathematical nature (universality) of the fundamental object of the universal thermal sum (10). This is it, traditional thermodynamics could not be made independent of the material specific physical values. In our formalism, this dependence is reduced to the minimum, i.e. to a single value $T_0$. However, even this value could be avoided if one knew atomic and elastic properties of a material. In fact, the ultimate form of scaling could be observed in liquids because they are truly isotropic and (usually) possess only a pressure velocity of sound. This subject will be worked out next, and the completion of the theory of specific heat for crystalline matter is postponed, because it requires a better expertise in crystallography.

The scaling postulate (19) might look odd at first, but in fact it is the most natural and common way of producing physical equations. Indeed, any fundamental equation in theoretical physics, from the Newton’s law of gravitation to the Dirac equation, contains dimensional parameters, which are called the physical constants. They match the mathematical structure of a physical theory to experimental measurements. In traditional thermodynamics of gases, the physical constant is the universal gas constant, *R*^22^, discovered and measured by Mendeleev^66^. Within the proposed thermal theory, *R* is universal only for gaseous matter. For condensed matter systems, there exist a number of thermal constants with the same physical dimensionality, each for every crystal lattice class, and the multitude of them for various glassy types.

The proposed theory resembles the theory of thermodynamic ensembles of Gibbs^24^. Traditional thermodynamics is also an axiomatic theory and its energy-like potentials are not directly connected to physical observables. Presented is a different axiomatic theory, based on a different branch of mathematics. Despite a similarity, the sum (10) is quite different from the sums of traditional thermodynamics. On one hand, no notion of energy is used or appear in (10), on the other hand, $\alpha ^2$ in the exponents of $F (\alpha )$ is proportional to the square of a sound velocity, thus, it could be associated with the kinetic energy of elastic waves in matter. We hypothesize that the sum (10) could explains the origin of the sums over ensembles in Gibbs’ theory, further investigation of this intriguing similarity is under way.

The way to complete the field theory of specific heat, in our opinion, is to replace a scalar function of temperature by the tensor quantity. Indeed, as long as temperature got connected with a velocity of sound, it became a vector, because velocity is a vector. In anisotropic matter, the stress tensor defined by elastic moduli gives the tensor of velocities. The possible way to relate the new tensor $\beta _{ij}$ with the temperature of traditional thermodynamics is to make some averaging, e.g. the determinant. Whether this method would be working could be seen through building specific models and calibrating them them with experimental data.

We eliminated physical time in the theory, since temperature, *T*, is expressed now with the inverse of the closed Euclidean time^4^. This is a natural step, because we want to describe phenomenology of what is usually called ‘thermal systems in equilibrium’. This step should be modified if we want to describe time-dependent phenomena, like non-stationary heat conductivity. At the same time, physical time is *implicitly* present in the theory through the velocity of sound, *v*, in its variable (18).

Over the last two decades extensive measurements the specific heat of glasses studied their universal thermal behaviour, in particular, the convex shape of $C/T^3$ function, like Fig. 7, was dubbed as the ‘boson peak’^67^. The main idea of our work is that this universal scaling can be explained by the universal function. The specific heat functions of glasses are qualitatively similar to crystals, i.e. their fundamental function is the same (13). Experimental evidence forces us to discard the old belief that glasses have thermal properties different in principle from those of crystalline matter, e.g.^68^. The observed quantitative difference is explained by the isotropy of glasses and anisotropy of crystals. Consequently, glasses possess only two, longitudinal and transverse, elastic waves, while crystals spectra are quasi-discrete, i.e. have several pronounced peaks, e.g.^58,69^. These differences are further complicated by the surface heat capacity. Thus, it is quite natural to first complete the theory for amorphous matter, before proceeding to crystalline one. We hope that experts on the physics of glasses would utilize the proposed mathematical ideas.

Since physics aims to discover the most general properties of natural phenomena, its theories must embody the most general mathematics. There is nothing more general and fundamental in mathematics than the geometry of spacetime, which could be explored with the versatile tool of the evolution equation^4^. The phenomenon of scaling, "Scaling in the specific heat data of the diamond lattice elements and the zincblend compounds" section, discovered empirically and mathematically, is clearly one of the most fundamental properties of Nature. This discovery should be investigated and elaborated into practical models that would aid engineers and technologists with analytical tools instead of data tables and numerical simulations.

## Data Availability

The 3rd party data, obtained in the experimental study described in Ref.^47^, which we analyze in the present paper, are made publicly available as Supplementary material with the permission of Prof. Reinhard Kremer. They can be used with the proper citation to Ref.^47^.
